# Virtual electrodes around anatomical structures and their roles in defibrillation

**DOI:** 10.1371/journal.pone.0173324

**Published:** 2017-03-02

**Authors:** Adam Connolly, Edward Vigmond, Martin Bishop

**Affiliations:** 1 Division of Imaging Sciences and Biomedical Engineering, King’s College London, St. Thomas’ Hospital, London, United Kingdom; 2 IHU Liryc, Electrophysiology and Heart Modeling Instituté, fondation Bordeaux Université, F-33600 Pessac-Bordeaux, France; 3 Univ. Bordeaux, IMB, UMR 5251, F-33400 Talence, France; University of Minnesota, UNITED STATES

## Abstract

**Background:**

Virtual electrodes from structural/conductivity heterogeneities are known to elicit wavefront propagation, upon field-stimulation, and are thought to be important for defibrillation. In this work we investigate how the constitutive and geometrical parameters associated with such anatomical heterogeneities, represented by endo/epicardial surfaces and intramural surfaces in the form of blood-vessels, affect the virtual electrode patterns produced.

**Methods and results:**

The steady-state bidomain model is used to obtain, using analytical and numerical methods, the virtual electrode patterns created around idealized endocardial trabeculations and blood-vessels. The virtual electrode pattern around blood-vessels is shown to be composed of two dominant effects; current traversing the vessel surface and conductivity heterogeneity from the fibre-architecture. The relative magnitudes of these two effects explain the swapping of the virtual electrode polarity observed, as a function of the vessel radius, and aid in the understanding of the virtual electrode patterns predicted by numerical bidomain modelling. The relatively high conductivity of blood, compared to myocardium, is shown to cause stronger depolarizations in the endocardial trabeculae grooves than the protrusions.

**Conclusions:**

The results provide additional quantitative understanding of the virtual electrodes produced by small-scale ventricular anatomy, and highlight the importance of faithfully representing the physiology and the physics in the context of computational modelling of field stimulation.

## 1 Introduction

The conventional strategy for termination of lethal cardiac arrhythmias such as fibrillation, by Implantable Cardioverter Defibrillators (ICDs), is to discharge a high-power capacitor through electrodes implanted in and around the heart. High-energy shocks are thought to activate excitable regions of tissue, thereby removing the path through which fibrillation wavefronts propagate, leading to the termination of the arrhythmia [1]. However, the high-energies required to defibrillate may rapidly deplete the ICD power reserves, whilst inappropriate shock therapies may cause serious physical pain, damage and psychological stress to the patient and increase mortality rates [2].

Recently, researchers have focussed on developing novel strategies for defibrillation that require a fraction of the energy of standard shock treatments [3–6]. The current understanding is that these techniques are driven by the “Virtual Electrode” (VE) effect, in which conductivity heterogeneities cause localised regions of depolarisation (and adjacent hyper-polarisation). In contrast to standard high-energy defibrillation where (it can be assumed that) the magnitude of the electric field is sufficiently large such that all heterogeneities emit wavefronts, Low-Energy Defibrillation (LED) relies on the emission of wavefronts only from heterogeneities with sufficiently intense VE depolarizations, which are assumed to be distributed spatially in the myocardium [4]. The mechanism of LED relies on there being one or more VEs with sufficiently close proximity to a spiral wave core such that, upon appropriately timed stimuli [7], wavefronts emitted by the VEs act to extinguish the spiral wave by exciting excitable gaps; effectively removing the spiral wave substrate or progressively pushing the spiral core towards the tissue boundary thereby terminating it [4, 8]. Standard defibrillation, in contrast, relies on the radically different post-shock wave-propagation activity to annihilate the phase-singularities responsible for the defibrillation.

Successful LED has been recently demonstrated in canine experiments during both atrial [3, 4, 6] and ventricular [4] fibrillation, in addition to ventricular tachycardia (VT) [6]. A recent *in-silico* study also showed that ventricular fibrillation (VF) could be terminated by a novel two-stage low energy protocol within a structurally-detailed model of the rabbit RV [5]. In addition to differing shock protocols, these studies also suggest that different physiological structures are primarily responsible for the success of LED [4, 5].

In the Luther *et al*. study, intramural blood vessels were suggested to be the dominant VE source, providing a mechanism for activation of the bulk myocardium. However, in contrast to the arguments made in [4], the Ranter *et al*. [5] modelling study showed that initial wavefront propagation occurred at points on the endocardial ventricular surface, before activation from VEs coincident with blood-vessels. Specifically, the simulations identified endocardial trabecular grooves (regions of positive curvature) as important sites of VE induction under weak field strengths. In contrast to this finding, a recent study [9] using idealised representations of anatomical structures suggested that VE wavefronts are more likely to arise on surfaces of negative curvature (such as endocardial trabeculation protrusions).

In this work we aim to clarify the basic understanding of the mechanisms by which VEs are formed by different anatomical features. As VE formation is known to be driven by heterogeneity in conductivity, we consider not only geometrical heterogeneity, but also pay attention to physiological conductivities and their heterogeneity in the structures considered. Using a combined analytical and numerical approach, we investigate the interaction of externally-applied electric fields with the coronary vasculature and surfaces of positive/negative curvature (representing endocardial grooves/protrusions). The magnitudes of the resulting induced VEs are analysed and the dependence of these effects upon structural and conductivity variations are uncovered.

In the ensuing analysis, we assume the myocardium is described well by the bidomain equations. We seek, whenever possible, analytical expressions for the VE field, as a thorough physical understanding of the parameters influencing the VE patten is a consequence of having an analytical description of the effects on the transmembrane potential due to applied electric fields.

## 2 Methods

### 2.1 Governing equations

The steady-state bidomain equations [10] are used to describe the transmembrane potential in the myocardium (Ω_*t*_), with Laplace’s equation describing the potential distribution in the bath (Ω_*b*_).

$$\begin{array}{c} \begin{array}{cc} \nabla \cdot (\sigma_{i}\nabla \phi_{i}) & =\beta \frac{V_{m}}{R_{m}},\;\in \Omega_{t}\\ \nabla \cdot (\sigma_{e}\nabla \phi_{e}) & =-\beta \frac{V_{m}}{R_{m}},\;\in \Omega_{t}\\ \sigma_{b}\nabla^{2}\phi_{b} & =0,\;\in \Omega_{b}. \end{array} \end{array}$$ (1)

Here *ϕ*_*i*_ and *ϕ*_*e*_ are the intra and extra-cellular potentials, *V*_*m*_ = *ϕ*_*i*_ − *ϕ*_*e*_ is the transmembrane potential, ***σ***_*i*_ and ***σ***_*e*_ are the intra and extra-cellular conductivity tensors, *σ*_*b*_ is the bath conductivity, *β* = 0.14 *μ*m^−1^ is the membrane surface area to volume ratio and *R*_*m*_ = 1.0 Ωm^2^ is the membrane resistance.

Eqs (1) are subject to the following boundary conditions, where ∂Ω_*t*_ is the boundary of the myocardium, ∂Ω_*tb*_ is the interface between the myocardium and the bath and ∂Ω_*b*_ is the boundary of the bath:

$$\begin{array}{c} \begin{array}{cc} n\cdot (\sigma_{i}\nabla \phi_{i}) & =0,\;\partial \Omega_{t},\partial \Omega_{tb}\\ n\cdot (\sigma_{e}\nabla \phi_{e}) & =\sigma_{b}n\cdot \nabla \phi_{b},\;\partial \Omega_{tb}\\ \phi_{e} & =\phi_{b},\;\partial \Omega_{tb}\\ \sigma_{b}n\cdot \nabla \phi_{b} & =0,\;\partial \Omega_{b}. \end{array} \end{array}$$ (2)

### 2.2 Approximations

In order to derive analytical expressions for the transmembrane potential *V*_*m*_, suitable approximations for the bidomain Eqs (1) are required.

#### 2.2.1 Approximation for isotropic tissue

When the effects of anisotropy may be reasonably neglected, the bidomain equations reduce to the isotropic monodomain equations, supplemented by Laplace’s equation for the bath-potential:

$$\begin{array}{c} \begin{array}{cc} \nabla^{2}V_{m}-V_{m}/\lambda^{2} & =0,\;\in \Omega_{t}\\ \left(\sigma_{i}+\sigma_{e}\right)\nabla^{2}\phi_{e} & =0,\;\in \Omega_{t}\\ \sigma_{b}\nabla^{2}\phi_{b} & =0,\;\in \Omega_{b}, \end{array} \end{array}$$ (3)

where *λ*^2^ = *R*_*m*_*σ*_*i*_*σ*_*e*_/(*β*(*σ*_*i*_ + *σ*_*e*_)) is the space-constant. Eqs (3) assumes that perturbations to ∇*ϕ*_*e*_ from the induced transmembrane potential (in response to field-stimulation) are small, such that they may be neglected, and assumes a parallel combination of the intra and extracellular isotropic conductivities for Laplace’s equation in the extracellular space [11, 12].

The isotropic tissue conductivities were computed from the harmonic mean of the experimentally measured [13] longitudinal and transverse conductivities, giving values of *σ*_*i*_ = 0.017 and *σ*_*e*_ = 0.173 S/m.

#### 2.2.2 Approximation for anisotropic tissue

When VEs form in response to anisotropic intra and extracellular conductivities, the perturbation scheme proposed in [14] is used, along with an analytical description of the fibre-field *ψ*. As lower order terms dominate, the perturbation expansion for the transmembrane potential is truncated to first-order:

$$\begin{array}{c} V_{m}=V_{m}^{0}+\epsilon V_{m}^{1}+\ldots , \end{array}$$ (4)

where *ϵ* = 1 − (*σ*_*el*_/*σ*_*et*_)/(*σ*_*il*_/*σ*_*it*_) and the subscripts *l*, *t* indicate the longitudinal and transverse directions, respectively. The zeroth-order contribution ($V_{m}^{0}$) is identical to the first equation in Eqs (3) and the first-order contribution comes from Equation (22) in [14]:

$$\begin{array}{c} \begin{array}{cc} V_{m}^{1} & \approx E_{0}\lambda^{2}\left[\text{cos}\left(\phi \right)\left(\text{sin}\left(2\psi \right)\psi_{x}-\text{cos}\left(2\psi \right)\psi_{y}\right)\right.\\ & \;\left.-\text{sin}\left(\phi \right)\left(\text{cos}\left(2\psi \right)\psi_{x}+\text{sin}\left(2\psi \right)\psi_{y}\right)\right], \end{array} \end{array}$$ (5)

where *ϕ* is the orientation of the externally imposed electric-field of strength *E*_0_ and subscript *x* or *y* indicates the partial derivative in the respective direction. Note that this approximation for $V_{m}^{1}$ assumes the electric field is a constant field, and not influenced by the different conductivities of the myocardium and bath.

### 2.3 Geometrical models

**Fig 1:**
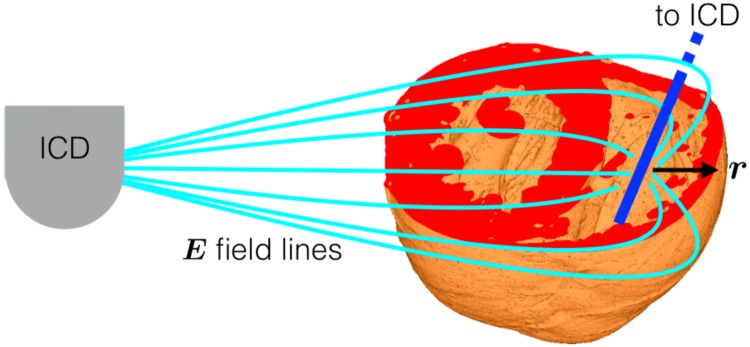
Schematic of intracardiac RV electrode (blue), with an active “can” far from the RV electrode. The electric field around the RV electrode is shown in light-blue.

The ICD electrode is often placed in the right-ventricular cavity with the shock-vector between it and the active “can”, creating an electric field around the electrode in the radial ***r*** direction, as shown schematically in Fig 1. Taking a cross-sectional slice, perpendicular to the apex-base direction, serves to create a two-dimensional representation of the myocardium which provides a simplifying assumption, facilitating the study of VEs around the specific geometrical features.

#### 2.3.1 Endocardial trabeculations

**Fig 2:**
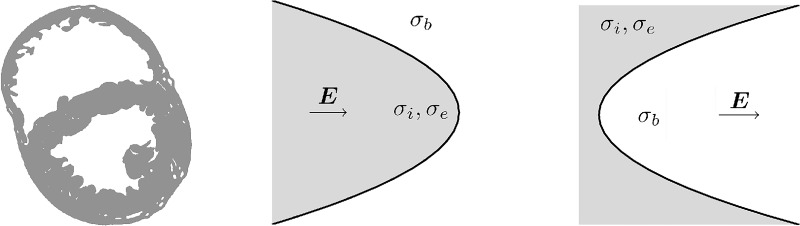
Cross-sectional slice of the rabbit ventricles [15] showing a highly trabeculated endocardial surface (left) and idealized geometries representing protrusions (middle) and grooves (right). The origin is located at the vertex of the parabolas.

The ventricular endocardial surface has typically many trabeculae carneae, which serve to keep the heart contracting in an optimal fashion. A cross-sectional MR image [15], in the axial plane, of the rabbit ventricles (shown by the left-hand pane in Fig 2), shows many trabeculae in both chambers. In order to study how the trabeculae are affected by far-field electrical stimulation, a single trabeculation protrusion or groove is idealized to form a parabolic boundary with the far-field stimulus aligned with the axis of the parabola (corresponding to the radial field-lines around the RV ICD electrode), as shown in Fig 2. The parabolic geometry necessarily implies that the stimulating electrodes are set at ±∞ in the x-direction which is of course unphysical; however this was done in order to allow direct comparison with results in [9]. As in [9], the curvature at the vertex of the parabola (defined as *x*(*y*) = *cy*^2^) may be varied by varying the factor *c*.

#### 2.3.2 Blood-vessels

**Fig 3:**
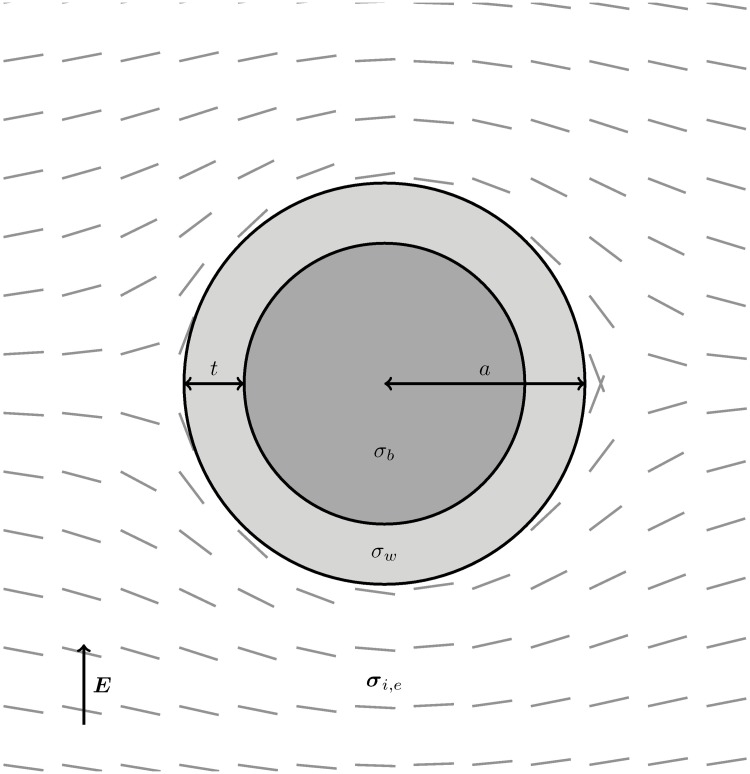
Blood-vessel schematic. The fibre-field (given by Eq (6)) is shown by the grey lines and the stimulating field is aligned with the vertical axis (corresponding to the field-lines around the RV ICD electrode in Fig 1).

Blood-vessels have a complicated and highly specialized structure, with the blood-vessel wall consisting of an elastic collagenous membrane with a surrounding muscular annulus which may provide tension to constrict or relax to dilate the vessel. The largest blood-vessels run in the apex-base direction [16] with myofibres smoothly circumnavigating the vessel [17]. Close to the blood-vessel, the bulk curvature of the ventricular wall may be neglected, and a reasonable description of the fibre-field is given by the unit-vectors of a potential flow around a circular cylinder [18] (with its axis co-located with that of the blood-vessel):

$$\begin{array}{c} \psi \left(r,\theta \right)={\text{tan}}^{-1}\left(\frac{2a^{2}\text{cos}\left(\theta \right)\text{sin}\left(\theta \right)}{2a^{2}{\text{cos}}^{2}\left(\theta \right)-r^{2}-a^{2}}\right), \end{array}$$ (6)

where *ψ*(*r*, *θ*) gives the fibre angle (the angle of the unit-vector of the potential flow). Fig 3 shows a schematic of a blood-vessel with wall-thickness *t* and outer radius *a*.

## 3 Results

### 3.1 Endocardial trabeculation VEs

In order to make the analysis tractable, the effect of fibre-curvature in the trabeculae is ignored (the tissue is assumed to be isotropic) and the governing equations for the myocardium reduce to Eqs (3). This is a reasonable assumption as surface VEs are induced by the transit of current into and out of the extracellular space only, and fibres close to the surface are generally tangent to the surface.

The semi-analytical scheme described in [9] may be used to solve the monodomain equation in the parabolic protrusion, however a numerical scheme must be used to solve the monodomain equation for the parabolic groove; in this work the FEM was used, with the no-flux condition imposed on rectangular boundaries far from the vertex. In both (groove and protrusion) cases, the monodomain boundary condition (***n*** ⋅ ∇*V*_*m*_ = −***n*** ⋅ ∇*ϕ*_*e*_) on the surface of the parabola was specified by knowing *ϕ*_*e*_. With a knowledge of *ϕ*_*e*_ and its gradient, the semi-analytical [9], or the FEM, scheme may then be used to compute the transmembrane potential.

The extracellular potential *ϕ*_*e*_ may be computed by solving Laplace’s equation via a conformal mapping to the parabolic coordinate system, with *x* = (*u*^2^ − *v*^2^)/2 and *y* = *uv*; this choice of coordinates allows the boundary of the parabola to lie on a constant ordinate *u* = *u*_0_. The effect of conductivities is to scale the current-flux through the parabolic surface by the factor *u*_0_*κ*/*h*, where *κ* = (*σ*_*i*_ + *σ*_*e*_)/*σ*_*b*_ for the protrusion or the inverse (*κ* = *σ*_*b*_/(*σ*_*i*_ + *σ*_*e*_)) for the groove (here $h=\sqrt{u_{0}^{2}+v^{2}}$ is the scale factor for the coordinate system and *u*_0_ = (2|*c*|)^−1/2^). Assuming *κ* = 1 gives a uniform field for both the groove and the protrusion, and relies on *σ*_*b*_ = *σ*_*i*_ + *σ*_*e*_, which is unphysiological.

**Fig 4:**
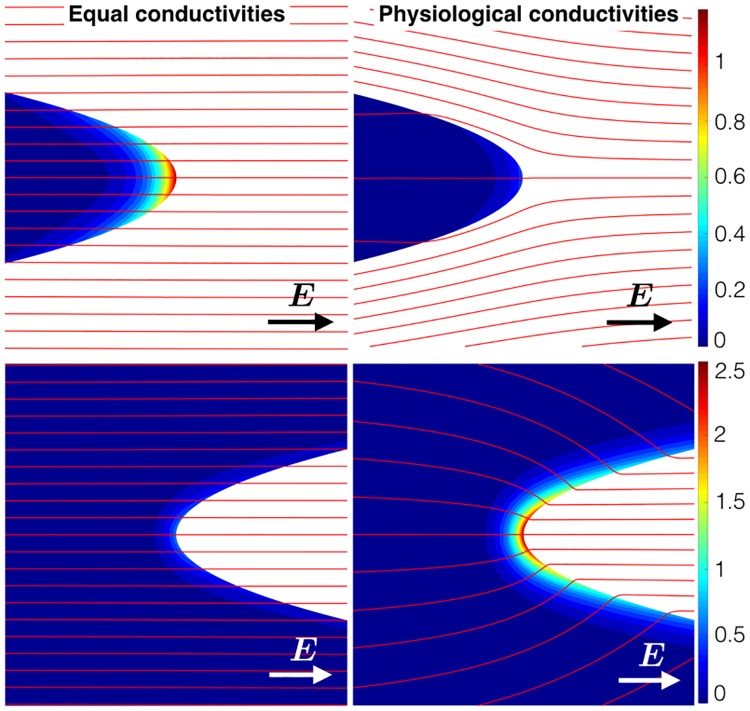
The non-dimensional transmembrane potential for the parabolic geometries with similar conductivities (left panel) and realistic conductivities (right-panel). The red lines show the streamlines of ***E***. Note that here the transmembrane potentials for the protrusions (top row) were calculated using the semi-analytical scheme [9], whereas the FEM scheme (solving the full bidomain system) was used to calculate the transmembrane potentials for the grooves (bottom row).

Fig 4 shows the change of the normalized transmembrane potential around its resting value in the region of the vertex, for parabolic boundaries of curvature *cλ* = ±0.4, for physiological conductivities *κ* ≈ 0.19 and similar conductivities *κ* = 1.0.

**Fig 5:**
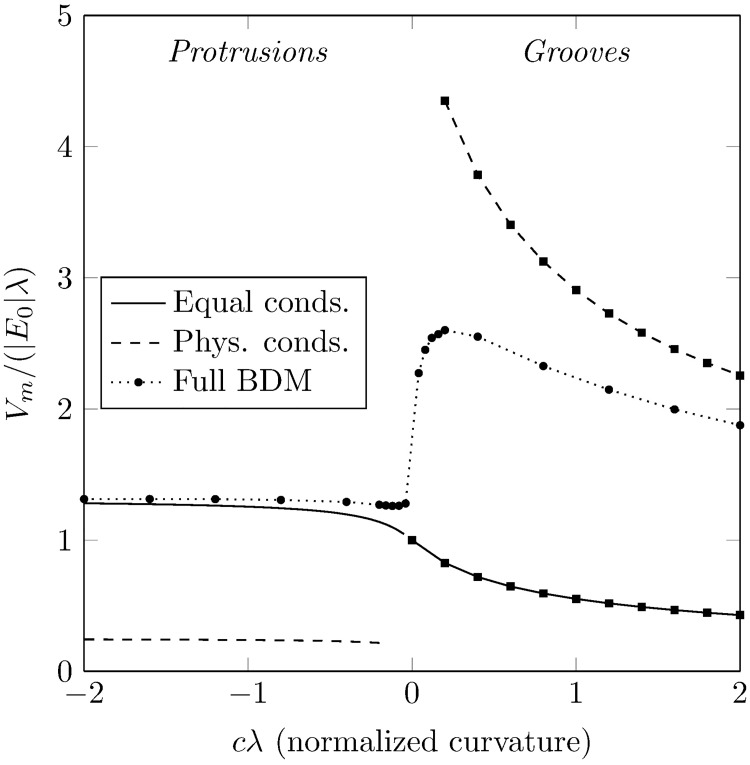
The normalized depolarization (measured at the vertex) of domains with parabolic boundaries versus non-dimensional curvature. Data-points for positive curvatures (*cλ* > 0) were calculated using unstructured linear finite elements. Two sets of curves, corresponding to equal conductivities (solid line) and physiological conductivities (dashed line), are shown. Full bidomain results in a truncated domain are also shown (dotted line).

The non-dimensional transmembrane potential, as a function of the normalized curvature, at the vertex of the parabola is shown in Fig 5. The discontinuity at *cλ* = 0 is due to the specific (infinite) geometry considered. To check this behaviour, we numerically computed (using CARP [19]) the full steady-state bidomain Eqs (1), with physiological conductivities, in a square domain of (*x*, *y*) ∈ *λ*([−40, 40] × [−40, 40]) with the tissue contained in *x* < *cy*^2^ and line electrodes at |*x*| = 40*λ*. We observed a jump in the maximum depolarization between small values of curvature, giving a continuous curve for *V*_*m*_ around *cλ* = 0, albeit with a large gradient (see the dotted curve in Fig 5). We also confirmed that increasing the size of the domain brought the maximum depolarization at the vertex of the parabolas closer to the semi-analytical results (dashed curves in Fig 5). Each of the curves in Fig 5 are discussed in sequence:

#### Equal conductivities, negative curvature

The effect of decreasing negative curvature is to increase the transmembrane potential relative to its value at *cλ* = 0; this is due to increasing confinement from the boundary [9]—the transmembrane potential may diffuse only in the domain of the tissue and in the limit *cλ* → −∞ the tissue becomes one-dimensional.

#### Equal conductivities, positive curvature

The effect of increasing positive curvature is to decrease the transmembrane potential relative to its value at *cλ* = 0; this is due to decreasing boundary confinement—the transmembrane potential may diffuse in the *x* direction only at *cλ* = 0 and as *cλ* → ∞ the diffusion may occur in both *x* and *y* directions. In effect, at *cλ* = ∞, the response of the tissue is similar to a point transmembrane stimulation in an infinite 2D sheet.

#### Physiological conductivities, negative curvature

The same boundary confinement occurs as in equal conductivities, raising the transmembrane potential relative to its value as *c* → 0^−^, however the magnitude of the boundary flux is much reduced due to current preferring to travel through the higher-conductivity bathing medium, reducing the induced transmembrane potential.

#### Physiological conductivities, positive curvature

The same effect of decreasing boundary confinement, lowering the transmembrane potential relative to its value as *c* → 0^+^, as in the case of equal conductivities. However, due to current preferring to travel through the higher-conductivity bathing medium, the current-density is relatively much larger at the vertex of the parabola resulting in a higher transmembrane potential.

### 3.2 Vessel VEs

In the ensuing analysis, we first impose a constant electric field in order to elicit the overall behaviour of the VE formed due to field stimulation. We then calculate, numerically, how the electric field varies around the blood-vessels to better understand the behaviour of the induced VE.

As it is known that the effects of fibre-curvature play an important role in the formation of VEs around blood vessels [20, 21], the approximate perturbation scheme [14] may be used to study the induced VEs.

It was assumed that the special fibre-architecture has a negligible effect on the zeroth-order component, and the resulting PDE (first equation of Eqs (3)) was solved [22] to give

$$\begin{array}{c} V_{m}^{0}\left(r,\theta \right)\approx -E_{0}\lambda \frac{K_{1}\left(r/\lambda \right)}{K_{1}^{\prime }\left(a/\lambda \right)}\sin\left(\theta \right), \end{array}$$ (7)

where *λ* is the space-constant and K_*ν*_ is the modified Bessel function of second-kind. The first-order contribution (Eq (5)) is given as

$$\begin{array}{c} \begin{array}{cc} V_{m}^{1}\left(r,\theta \right) & \approx -\frac{E_{0}\lambda^{2}}{r}\left[\text{sin}\left(\psi \right)\left(\psi_{\theta }\left(2{\text{sin}}^{2}\left(\psi \right)-1\right)+2\psi_{r}r\text{cos}\left(\psi \right)\text{sin}\left(\psi \right)\right)\right.\\ & \;+\left.\text{cos}\left(\psi \right)\left(2\psi_{\theta }\text{cos}\left(\psi \right)\text{sin}\left(\psi \right)+\psi_{r}\left(r-2r{\text{sin}}^{2}\left(\psi \right)\right)\right)\right],\\ & \approx -\frac{E_{0}\lambda^{2}}{r}\left[\frac{\text{sin}\left(\theta \right)\left(\text{cos}{\left(\theta \right)}^{2}\left(8a^{8}+8a^{2}r^{6}\right)-2a^{2}{\left(a^{2}+r^{2}\right)}^{3}\right)}{{\left(4a^{2}r^{2}\text{sin}{\left(\theta \right)}^{2}+{\left(a-r\right)}^{2}{\left(r+a\right)}^{2}\right)}^{2}}\right]. \end{array} \end{array}$$ (8)

#### 3.2.1 VE polarity swapping

**Fig 6:**
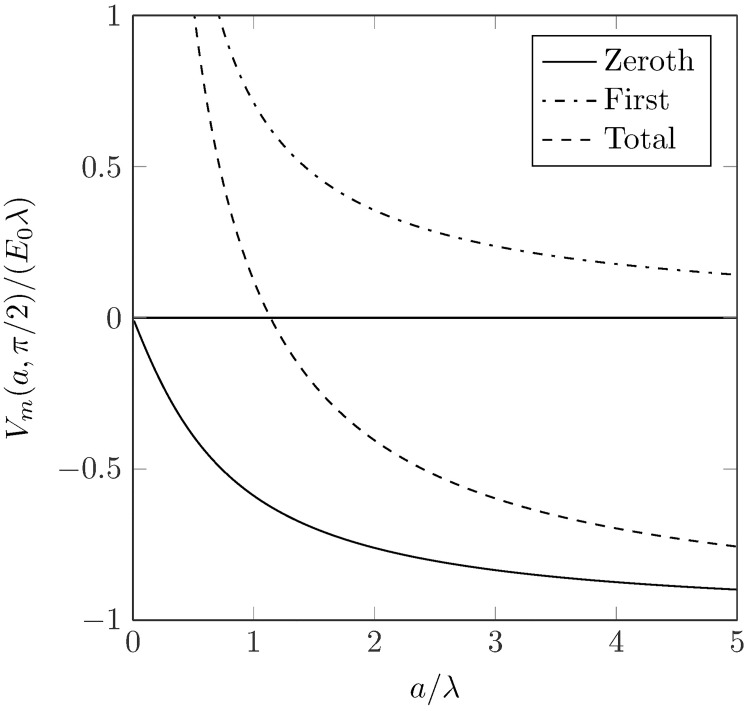
The value of the normalized VEs at *r* = *a*, *θ* = *π*/2 for the zeroth-order component (solid line), the first-order component (dot-dashed) and the resultant potential (dashed).

**Fig 7:**
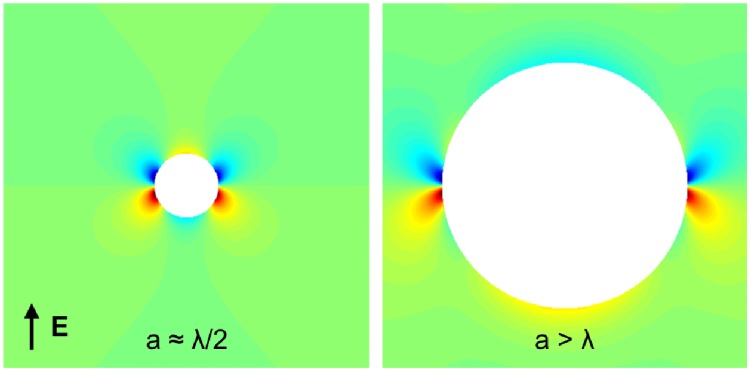
The VE pattern around a blood-vessel with an electric field aligned with the *y* axis. Red and blue colours indicate depolarization and hyperpolarization, respectively.

Evaluating the approximate transmembrane potential (from Eq 4) on the surface of the vessel at *r* = *a*, *θ* = *π*/2 gives

$$V_{m}\left(a,\pi /2\right)\underset{˜}{\propto }-\frac{{\text{K}}_{1}\left(a/\lambda \right)}{{\text{K}}_{1}^{\prime }\left(a/\lambda \right)}+\epsilon \frac{\lambda }{a},$$ (9)

where the first and second terms on the r.h.s of Eq (9) correspond to the zeroth and first-order contributions to the transmembrane potential, respectively. Conversely, evaluating at *r* = *a*, *θ* = 3*π*/2 gives *V*_*m*_(*a*, 3*π*/2) = −*V*_*m*_(*a*, *π*/2). Fig 6 shows the relative contributions of the two components. It can be seen that, for some value of *a*, the resultant potential swaps signs. Qualitatively, when *a* is small, the second-term in Eq (9) dominates and when *a* is big, the first-term dominates. The transmembrane potential at *r* = *a* for *a* → 0 diverges due to the first-order component, however the approximation in Eqs (9) and (8) is only reasonable for radii of the order of the space-constant [14]. As *a* → ∞ the first-order component vanishes and the zeroth-order component reaches its asymptotic value—this is to be expected as the curvature of the vessel boundary vanishes and thus the fibre-field is spatially uniform. Fig 7 shows the VE patterns for *a* ≈ *λ*/2 and *a* > *λ*, showing the swapping of the signs. The physical effect of a smaller vessel radius is to lower the magnitude of the dipolar zeroth-order contribution, as less current is transiting the vessel surface due to the lower surface diameter. The next section investigates the effects of the realistic conductivities of the vessel components on the VE patterns produced.

It should be noted that the strong VEs around *θ* = 0, *π* in Fig 7 are due to the abrupt changes in fibre-orientation at these points (see Fig 3), meaning that the analytical VE predicted around *r* = *a*, *θ* = 0, *π* (Eq (8)) is highly inaccurate as the assumptions of smoothness in the fibre-field [14] are violated. However, around *r* = *a*, *θ* = *π*/2, 3*π*/2 the fibre-field is smooth and the VE prediction (Eq 9) is a reasonable approximation.

#### 3.2.2 VEs with realistic conductivities

The perturbation scheme used above is of limited utility when including the effects of realistic conductivities, as the simplifying assumption (that the stimulating field is uniform), is violated and thus, in order to proceed, the bidomain equations must be solved numerically; for this we used the Cardiac Arrhythmia Research Package (CARP) [19].

**Fig 8:**
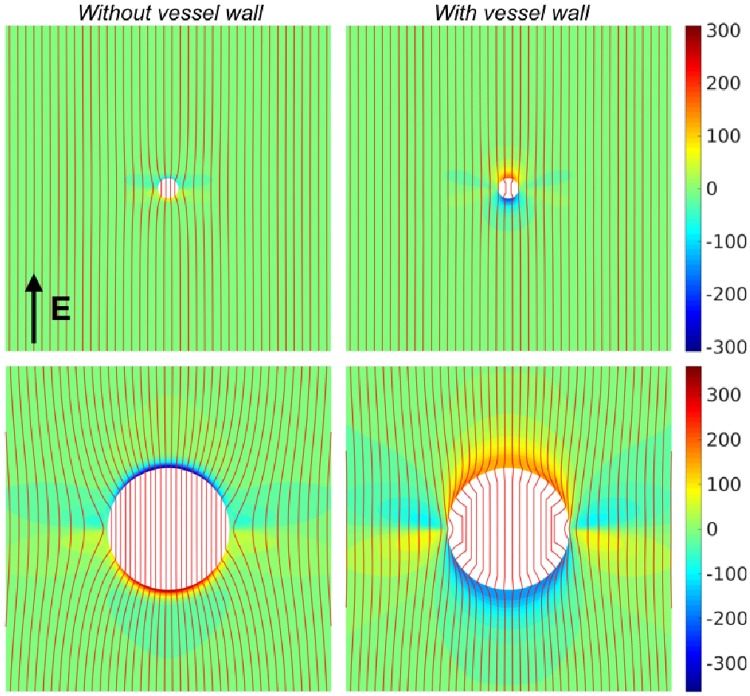
The steady-state bidomain solution for the transmembrane potential around blood-vessels of radius 0.5 mm (top row) and 3.0 mm (bottom row). The columns show the effects of the conductivities of the vessel cavity, in the case of no vessel wall (left column) and with a vessel wall (right column). The externally applied electric field was aligned with the vertical axis and had strength 10 V/cm; red lines show the streamlines of the electric field. The colorbar units are millivolts.

The tissue and vessel components were assigned the physiologically realistic conductivities (in S/m) of ***σ***_*i*_ = (0.17_*l*_, 0.019_*t*_) (intracellular myocardium) and ***σ***_*e*_ = (0.62_*l*_, 0.24_*t*_) (extracellular myocardium) [13], *σ*_*w*_ = 0.01 [20] (vessel-wall) and *σ*_*b*_ = 1.0 [23] (blood inside vessel). The vessel-wall thickness *t* was a function of the vessel radius *a*, through *t* = 3.87*a*^0.63^ [24], corresponding to measured values for the human coronary vasculature. The steady-state bidomain solution for the resulting VE pattern is shown in Fig 8. Fig 8 shows how the VE pattern changes with and without a vessel wall. It can be seen that the magnitude of the resultant VE, for a small vessel with no vessel wall (upper-left), is relatively low compared with the case with a vessel wall (upper-right).

In the case of a large vessel, the magnitude of the VE is higher without a vessel wall (lower-left) than with a vessel wall (lower-right), however the VE is more localised to the immediate upper and lower surface of the vessel. The resulting patterns may be understood in terms of the relative contributions of the zeroth and first-order components of the transmembrane potential, from Fig 6, and from physical arguments. In the case of no vessel-wall, current prefers to travel through the surface of the blood-vessel through the relatively high-conductivity blood, which acts to lower the current-density in the myocardium immediately surrounding the vessel. This has the effect of raising the zeroth-order, and lowering the first-order, contributions to the transmembrane potential. In the case of a vessel with a low-conductivity vessel wall, the zeroth-order contribution is lowered (as current is effectively shielded from passing through the vessel cavity) and the first-order contribution is raised (as the current-density in the immediate vicinity of the vessel is raised). In this specific case, these effects conspire to change the sign of the VEs at *θ* = *π*/2, 3*π*/2, for the case with and without a vessel-wall (see Section 4.2 for an explanation for the polarity swapping between small and large vessels (both with a vessel wall) observed in [20, 21]).

The red streamlines highlight how the current flows around the blood-vessels. As can be seen in each of the panels in Fig 6, the current-density (proportional to the streamline density) inside the vessel cavity is higher for vessels without a vessel wall, relative to vessels with a vessel wall. In the case of a vessel-wall, the streamlines can be seen to diverge around the vessel, lowering the zeroth-order contribution to the transmembrane potential. This behaviour is analogous to the effect around endocardial protrusions (left-panel in Fig 4), where the relatively low conductivity of the tissue reduces the current-density exiting the tissue surface, lowering the surface VE.

**Fig 9:**
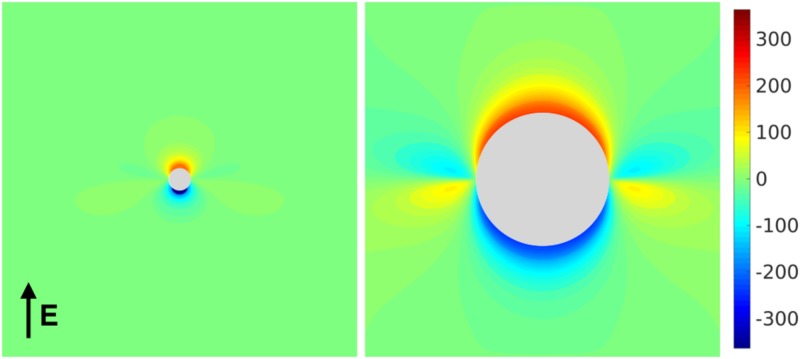
The steady-state bidomain solution for the transmembrane potential around insulators of radius 0.5 mm (left) and 3.0 mm (right). The externally applied electric field was aligned with the vertical axis and had strength 10 V/cm; red lines show the streamlines of the electric field. The colorbar units are millivolts.

In the limit that the conductivity of the blood-vessel wall vanishes, no current traverses the vessel surface causing the zeroth-order component of the transmembrane potential to vanish and the first-order hexapolar component to dominate. The VE around an insulating cylinder was observed experimentally using optical-mapping in [25]—it can be seen that the VE patterns shown in Fig 1 (B) in [25] are qualitatively similar to those presented in Fig 8 with the presence of an insulating vessel wall. We recomputed the VE field around perfect insulators to confirm that the VE pattern is indeed similar—see Fig 9.

## 4 Discussion and conclusions

In this study, we sought to provide a detailed mathematical and physical understanding of how applied electric fields interact with cardiac anatomical structures, driving the formation of VEs which are suggested to play an important role in successful defibrillation. We investigated how conductivity differences combine with the geometry to cause important local variations in the electric field that consequently affects the VE formation. Our analysis provided physical explanations for the preferential occurrence of secondary sources from endocardial grooves, in preference to both endocardial protrusions and blood vessels and also a detailed mathematical understanding of the specific VE patterns that occur around vessels and how these are governed by vessel size and wall structure.

### 4.1 Preferential wave propagation from endocardial trabeculation grooves

A major finding from this study is the importance of faithfully representing the bath space surrounding the myocardium, and the corresponding differences in physiological conductivities, in the context of field-stimulation. The different conductivities of the myocardium and blood effectively scale the magnitude of the VE induced by the trabeculation by the appropriate conductivity ratio. In the case of the trabeculae protrusion the induced VE has a reduced magnitude, however in the case of the trabeculae groove the induced VE has an increased magnitude (see dashed-curves in Fig 5). This suggests that wavefront propagation is more likely to occur in the grooves between the trabeculae protrusions; an effect which has been observed in bidomain simulations [5] of detailed MRI derived geometries.

Neglecting the physiological conductivities, or assuming that the conductivity of the myocardium and perfusing bath are equal (solid curves in Fig 5) implies that wavefront propagation is more likely to occur from trabeculae protrusions, as found in [9]. It is important to note here that this effect (wave propagation from surfaces of negative curvature) was experimentally verified in [9], using a neonatal myocyte monolayer preparation bathed in minimum essential medium. We digitized the geometry used in [9] and numerically solved the full bidomain equations (results not shown) with an active cell membrane and found that we were able to replicate the experimental findings [9] only when the tissue extracellular conductivity was similar to the bathing conductivity, which is reasonable for a monolayer preparation submerged under a thin layer of bathing medium [26, 27]. When the conductivity of the bathing medium was much higher than the conductivity of the myocardium (as per the physiological values), wavefront propagation preferentially occurred on surfaces of zero-curvature. The implication for numerical simulations of defibrillation is that it is important to solve Laplace’s equation for the bath-potential; full bidomain simulations generally include this step, however monodomain schemes often require a simplifying assumption regarding the behaviour of the electric field in the bath [9] which, as demonstrated here, may lead to different conclusions regarding the sites of initiation of wavefront propagation in response to far-field stimuli.

The finding that wavefronts preferentially initiate from the endocardial trabecular grooves at the lowest field strengths (with higher field strengths required to initiate from trabecular protrusions and vessels) suggests that novel low energy defibrillation protocols should exploit (through specific shock vector configurations) highly trabeculated ventricular regions in order to maximise secondary source formation; for example focussing the field more apically, and more within the RV. It also suggests the importance of including such details within ventricular models investigating such protocols, as local field strengths on smooth endocardial surfaces (from more anatomically simplified models, derived, for example from low resolution clinical MR data) will not produce the effects seen in trabecular grooves in this work. The importance of trabecular detail in the interaction with applied low energy fields may also explain potential interspecies differences in response to low energy defibrillation protocols, due to the known significant variation in trabeculation structure between different species [16]. Such differences may underlie potential issues in translating the successful pre-clinical studies performed primarily in canine preparations [3, 4, 6] into the clinic.

### 4.2 Vessel virtual electrodes

Previous modelling and experimental studies have suggested the importance of vessels in creating sources of excitation upon field stimulation [4, 20, 21, 25]. In this study, we showed how the contributions to the vessel VE are composed, mathematically, in terms of the zeroth and first-order contributions, and physically, from current transiting the vessel surface and current redistribution due to conductivity anisotropy, respectively. Importantly, our numerical results showed how the presence of a low-conductivity vessel-wall drastically alters the VE pattern. In particular, the results show how the presence of the insulating vessel wall reduces the magnitude of the dipolar surface VE, causing the polarity of the surface VE to swap (relative to the case with no vessel wall); an effect first noticed in [20, 21]. When the blood-vessel is represented by an internal boundary, on which the intra- and extracellular current-densities vanish (results not shown), the induced VE is similar to that induced in the case with an insulating vessel-wall, and consistent with experimental observations [25].

Note that, unlike the results presented here, in [20, 21] the polarity swapping occurred between small and large vessels, both with vessel walls. This was due to the specific way the the vessel-wall was represented in [20, 21]; a single layer of finite elements in contact with the vessel outer-radius, of roughly constant volume, were assigned a low conductivity to represent the vessel-lumen. This meant that the ratio of the vessel-wall thickness to vessel radius *t*/*r* varied (much more rapidly than the empirical function used in this work), such that, at large values of *r* the ratio *t*/*r* → 0 and the influence of the low-conductivity vessel wall was negligible, resulting in a dominant zeroth-order VE, changing the VE polarity.

The VEs around vessels suggest that the excitation strengths and mechanisms may be significantly different when the vessel-wall is taken into account, as the presence of a vessel wall causes VE depolarizations of similar magnitude and opposite sign to be relatively proximal to one another (compared with the VE pattern without a vessel wall). Successful defibrillation relies not only on initiating secondary sources to activate excitable tissue, but also on exciting tissue that is relatively refractory (which will regain excitability soon after the shock). Our analysis suggests that vessels may still play an important role in low-energy defibrillation due to the close proximity of de- and hyper-polarised regions around the vessel, which we have shown is augmented by the presence of the vessel wall. Such proximity of regions of opposite polarity may facilitate break excitations, similar to the mechanism for unipolar stimulation [28, 29], allowing vessels to capture intramural tissue that is relatively refractory.

As we have shown, VE strength is relatively raised at trabeculae grooves (compared to vessels), making them more likely sources of wavefronts upon field stimulation, for diastolic tissue. However, trabeculae VEs are generally of the same sign, thus they may not facilitate break excitations in a manner that vessels may.

Due to the vast extent of the vasculature network, our analysis suggests that vessels may thus still provide an important means of intramural secondary source formation during complex episodes of arrhythmias in which large parts of the tissue are relatively refractory. This finding may underlie previous suggestions of the importance of vessels in secondary source formation [4]; however, given the relatively higher field strengths required for break excitation formation [28, 29], such a vessel-mediated mechanism may only be of significance during stronger (conventional) defibrillation protocols, and less so during low voltage protocols.

#### 4.2.1 Modelling considerations for blood-vessels

From a modelling perspective, it may be difficult to physically resolve the blood-vessel wall, even in high-resolution models, as the thickness of the vessel-wall is necessarily much smaller than the vessel radius. Here we propose a compromise, which facilitates the inclusion of the effects of the blood-vessel wall, without resorting to physically resolving the blood-vessel wall in the computational model. Assuming the tissue is isotropic and the electric-field is oriented orthogonally to the axis of the vessel, and is uniform in the far-field, the conductivity of the vessel cavity may be modulated to approximate the effect of the blood-vessel wall *σ*_*w*_, the wall-thickness *t*, the conductivity of blood *σ*_*b*_ and the vessel radius *a*. Using the particular solutions for Laplace’s equation in polar coordinates, along with the electrostatic interface and boundary conditions, and solving for the case of two concentric cylinders (of radii *a* and *a* − *t*);

$$\begin{array}{c} \phi \left(r,\theta \right)=\left\{\begin{array}{cc} C_{1}r\text{sin}\left(\theta \right), & r\leq (a-t)\\ \left(C_{2}r+C_{3}/r\right)\text{sin}\left(\theta \right), & (a-t)\leq r\leq a\\ \left(-E_{0}r+C_{4}/r\right)\text{sin}\left(\theta \right), & r\geq a, \end{array}\right. \end{array}$$ (10)

where *C*_*x*_ are non-dimensional coefficients, allows to define a current-density at *r* = *a*. By equating this current-density with that obtained via the particular solution for one conducting cylinder of radius *a*, we can define an “equivalent conductivity” *σ*_*eq*_, which approximates the effect of physically resolving the blood-vessel wall. The equivalent conductivity is stated as

$$\begin{array}{c} \sigma_{eq}=\frac{t^{2}\sigma_{w}\sigma_{bw}+2at\sigma_{w}\sigma_{bw}+2a^{2}\sigma_{b}\sigma_{w}}{2at\sigma_{bw}-t^{2}\sigma_{bw}+2a^{2}\sigma_{w}}, \end{array}$$ (11)

where *σ*_*bw*_ = *σ*_*b*_ − *σ*_*w*_. Eq (11) may be used to specify the conductivity of computational elements/volumes inside blood-vessels, using the instantaneous radius *a* which may itself be computed using image thresholding and skeletonization operations similar to that used in [4].

As volumetric images of sufficiently high resolution to resolve the major coronary vasculature are not routinely captured in the clinic, it is proposed here that future work should focus on developing a robust scheme to superimpose a generic vasculature network, for the purposes of personalized bidomain modelling of defibrillation, in much the same way as myocardial fibre-architecture is superimposed using a rule-based algorithm [30, 31].

### 4.3 Suggested experimental validation

The effect of preferential wavefront initiation in endocardial grooves as opposed to protrusions may be experimentally tested for using optical mapping of the endocardial surface, with a field-stimulus oriented in the transmural direction. Validation of the results in this paper would require excitation (wavefront propagation) to occur preferentially in the endocardial grooves for the lowest field-strengths. Potentially, the conductivity of the bathing medium surrounding the preparation could also be varied, thus altering the magnitude the VEs and the locations of the wavefront initiations, as shown here.

Experimental validation of the bidomain prediction of VE patterns around intramural blood vessels is not possible with current epi-fluorescent optical mapping technologies due to the known issues of imaging transmembrane potential levels with sufficiently high resolution during shocks throughout the myocardial wall. Imaging cut transmural surfaces of ventricular wedge preparations or tissue slices may allow such measurements to be made, although such preparations often suffer from surface tissue damage and signals are inherently distorted due to photon scattering making the relatively small, highly contrasting, VE patterns around vessels problematic to resolve [32, 33]. One could, however, replicate the study in [25] (in which the VE around an insulating cylinder was optically mapped) and extract the insulating cylinder, leaving a cavity which may then be filled with high-conductivity fluid. Our results suggest that the sign of the VEs along the line parallel to the applied field, and intersecting the cylinder axis, will swap when the insulating cylinder is replace with highly conducting fluid.

It is important to note that a recent combined experimental and modelling study [34] cast doubt as to whether blood vessels are an important source of VE formation under weak field stimuli. Instead, they suggested that shock-induced “hot spots” of excitation originated from intramural sites which were not co-located with the vasculature, and which occurred at lower field-strengths than surface activations. Their suggestion that their findings were driven by other intramural structural/conductivity heterogeneities (other than vessels) requires further experimental investigation and have necessarily not been considered here as it challenges the bidomain theory of defibrillation (used in this work), which is a relatively mature and its predictions have been experimentally verified in numerous works [35].

## 5 Limitations

All the results presented are for steady-state, which correspond with very long stimulation pulses only. With shorter—more clinically relevant—stimulation pulses it may be expected that subtly different VE patterns may dominate the excitation behaviour as different parts of the VE field approach steady-state at different times.

## 6 Conclusions & clinical implications

Electrotherapy delivered via an ICD remains sub-optimal due to the high field strengths necessary to successfully terminate lethal cardiac arrhythmias. Understanding the interaction of electric fields with cardiac tissue is vitally important for optimising ICD electrotherapy and advancing novel low-energy approaches which have yet to translate clinically from recently suggested successful pre-clinical studies [3, 4, 6]. Our findings have demonstrated the importance of including fine-scale cardiac anatomical features, such as endocardial structures and intramural vessels, within computational models used for the further investigation of low-energy electrotherapy (and, indeed, standard defibrillation) which play a role in the development and refinement of such protocols. Furthermore, our results emphasise the importance of using a full bidomain model that fully represents the differing conductivity of surrounding bath (or blood), in order to faithfully represent the electric field at the tissue surface. Overall, the specific findings from this work may aid in the understanding of why the defibrillation efficacy changes between species and, potentially, between individuals (for example in different types of structural heart disease such as hypertrophic cardio-myopathy or hyper-trabeculation syndrome).
